# Molecular Diagnostics of Ciliopathies and Insights Into Novel Developments in Diagnosing Rare Diseases

**DOI:** 10.3389/bjbs.2021.10221

**Published:** 2022-01-10

**Authors:** K. Modarage, S. A. Malik, P. Goggolidou

**Affiliations:** Department of Biomedical Science and Physiology, Faculty of Science and Engineering, University of Wolverhampton, Wolverhampton, United Kingdom

**Keywords:** whole exome sequencing, cilia, rare disease, polycystic kidney disease, ciliopathies

## Abstract

The definition of a rare disease in the European Union describes genetic disorders that affect less than 1 in 2,000 people per individual disease; collectively these numbers amount to millions of individuals globally, who usually manifest a rare disease early on in life. At present, there are at least 8,000 known rare conditions, of which only some are clearly molecularly defined. Over the recent years, the use of genetic diagnosis is gaining ground into informing clinical practice, particularly in the field of rare diseases, where diagnosis is difficult. To demonstrate the complexity of genetic diagnosis for rare diseases, we focus on Ciliopathies as an example of a group of rare diseases where an accurate diagnosis has proven a challenge and novel practices driven by scientists are needed to help bridge the gap between clinical and molecular diagnosis. Current diagnostic difficulties lie with the vast multitude of genes associated with Ciliopathies and trouble in distinguishing between Ciliopathies presenting with similar phenotypes. Moreover, Ciliopathies such as Autosomal Recessive Polycystic Kidney Disease (ARPKD) and Meckel-Gruber syndrome (MKS) present with early phenotypes and may require the analysis of samples from foetuses with a suspected Ciliopathy. Advancements in Next Generation Sequencing (NGS) have now enabled assessing a larger number of target genes, to ensure an accurate diagnosis. The aim of this review is to provide an overview of current diagnostic techniques relevant to Ciliopathies and discuss the applications and limitations associated with these techniques.

## Introduction

**FIGURE 1:**
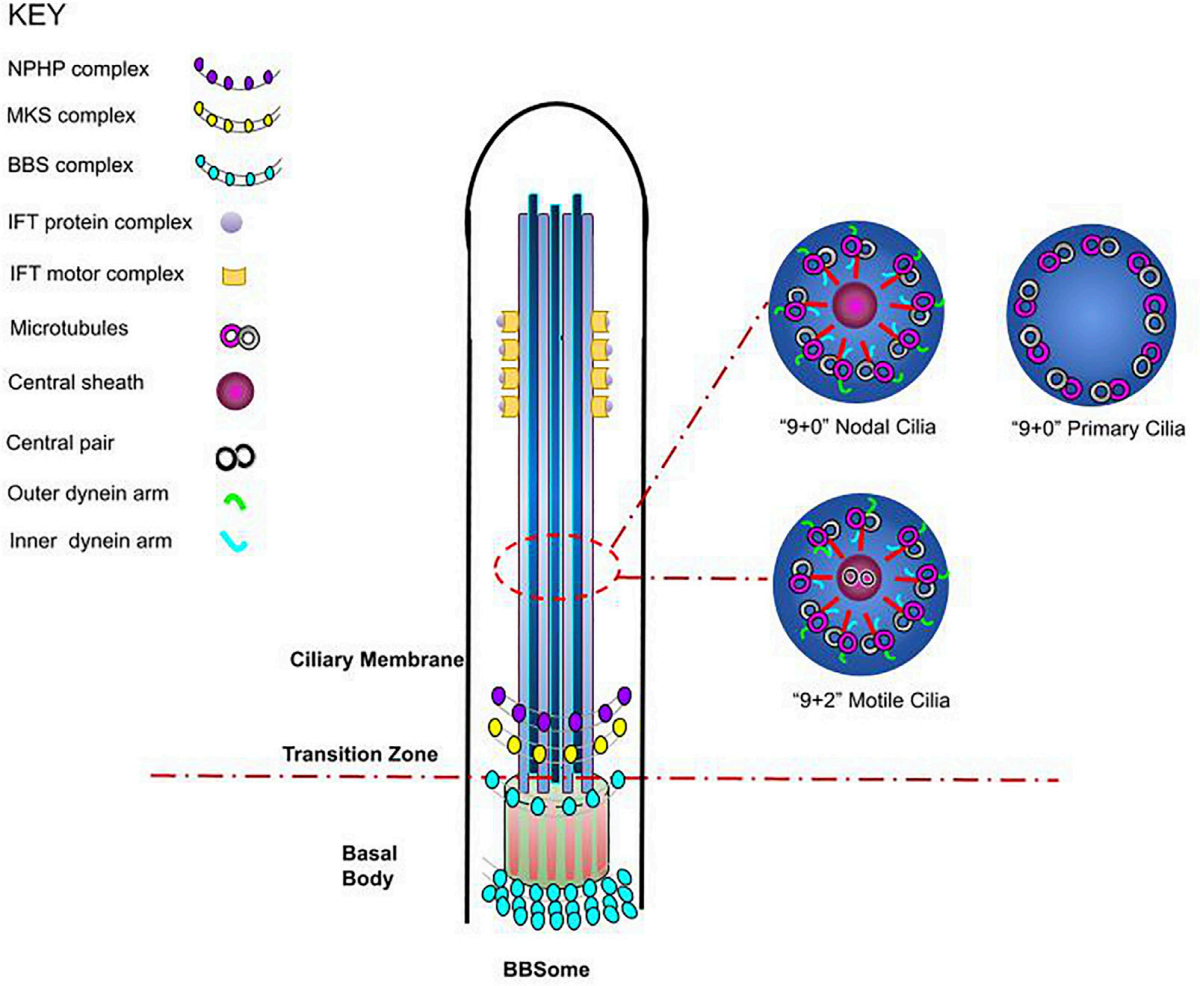
Schematic representation of cilium structure. The cilium is a membrane-bounded sensory organelle that is composed of the ciliary membrane, axoneme, and basal body. The ciliary membrane and axoneme form the upper region of the cilium, whereby nine peripheral microtubule doublets are present, whilst in the junction on the basal body, the transition fibres and key ciliary complexes are found^3,5^. In addition, a cross section of cilia axoneme illustrates the microtubule arrangements in nodal, motile, and non-motile (primary) cilia, highlighting the differences in cilium structure^3,5^.

Ciliopathies describe a group of disorders that arise due to mutations in cilia, resulting in their abnormal formation or function^1^. The term entails a group of around 35 reported disorders, with effects seen in multiple organs^1^. Cilia are short extracellular structures, projecting from the cell membrane that can be classified into motile or immotile (also known as primary) cilia^1^. Both motile and primary cilia begin to form during the G0/G1 phase of the cell cycle^2,3^. Motile cilia comprise of nine pairs of microtubules surrounding an additional central pair (9 + 2 arrangement) and present as multiple cilia per cell (Figure 1)^2,3^. They are usually longer than primary cilia and consist of specialised ciliary motor and accessory proteins, allowing the structure to actively bend and undergo coordinated beating patterns that create flow over the cell surface^4^. Primary cilia consist of microtubules arranged in a circular pattern, as nine microtubule doublets (9 + 0 arrangement)^5^; they play an essential mechanosensory role and have been implicated in various cell signalling pathways. One exception is the nodal cilium, a structure which presents with a 9 + 0 arrangement, however, displays directional movement^6^. In this review, the structure of the cilium will be broadly classified and discussed as two separate compartments – the base of the cilium and intraflagellar transport (IFT).

The basal body lies at the base of the cilium, where the ciliary gate is located (Figure 1). The ciliary gate is composed of three main structures: the transition fibres (TFs), the transition zone (TZ) and the ciliary necklace and it behaves as a docking site for IFT particles^1,5,6,7^. The gate is required for ciliogenesis by functioning as a diffusion barrier speculated to aid the transport of proteins and assembled IFT machinery through the formation of pore-like structures^1,5,7,8^; multiple TZ proteins have been identified to belong to either the Nephronophthisis (NPHP) or the Meckel Gruber Syndrome (MKS) complexes^9^. IFT has also been associated with the BBSome, where either independently or through cooperation with the BBSome, it is essential for the formation of the ciliary membrane (Figure 1)^1,10^. The BBSome is a complex of Bardet-Biedl syndrome (BBS) proteins, and it is yet to be established whether this structure is an additional constituent carried by IFT or an integral component of IFT machinery^11,12^. The correct formation of the above ciliary structural components is essential for their function, with mutations and defects in ciliary genes resulting in the onset of several Ciliopathies such as NPHP, MKS and BBS^1,5,7^. Since only a few disorders have a known molecular mechanism and there is overlap in the manifestation of many Ciliopathies, challenges are commonly faced when diagnosing these diseases.

## Historic and Current Diagnostic Approaches for Ciliopathies

Linkage analysis uses microsatellite markers for the analysis of genes under investigation and it has historically been a commonly practiced technique for the diagnosis of Polycystic Kidney Disease (PKD)^13^. In Autosomal Dominant Polycystic Kidney Disease (ADPKD), symptoms manifest between the ages of 30–50^1,14,15^ and include renal cysts, hypertension, gross haematuria, nephrolithiasis and urinary tract infections^1,14,15^. Extra-renal manifestations involve the development of hepatic, pancreatic and thyroid cysts and intracranial aneurysms^1,14,15^. Comparatively, ARPKD is common in neonates and infants^1,14^ and may manifest by bilateral enlargement of kidneys, the formation of fluid-filled cysts throughout the collecting ducts of the kidney, oligohydramnios, pulmonary hypoplasia, hepatic fibrosis, and respiratory insufficiency^1,14,15^. Defective genes associated with ADPKD include *Polycystic Kidney Disease 1/2 (PKD1/2)*, with *PKD1* accounting for most ADPKD cases^14^ (Table 1). In comparison, ARPKD has been associated with mutations in *Polycystic Kidney and Hepatic Disease 1 (PKHD1),* with recent findings linking mutations in *DAZ interacting protein 1-like (DZIP1L)* with moderate ARPKD^17^. *PKD1/2* and *PKHD1* encode for Polycystin (PC)-1/2 and Fibrocystin respectively, proteins that localise to the cilium^1,4,5,14,15^.

**TABLE 1:** A summary of selected Ciliopathies, including their associated genes and their current and recommended molecular diagnostic techniques.

Ciliopathy	Genes associated with ciliopathies	Current diagnostic practices	Novel molecular diagnostic practices recommended
Meckel-Gruber syndrome (MKS)	*MKS1,MKS3, MKS4, MKS5, MKS6, MKS7*	RT-PCR	TaqMan minor groove binder probes (Prenatal) whole-exome sequencing Whole-genome sequencing
Bardet-Biedl syndrome (BBS)	*BBS1, BBS2, BS10, MKS1, MKS4*	Clinical assessment	Targeted exome sequencing (Prenatal) whole-exome sequencing Whole-genome sequencing
Autosomal Recessive Polycystic Kidney Disease (ARPKD)	*PKHD1, DZIP1L*	Linkage analysis Direct mutation screening via amniocentesis and CVS	Whole-exome sequencing Whole-genome sequencing e.g., STATseq
Nephronophthisis (NPHP)	*NPHP1, NPHP20*	Direct mutation screening	Multiplex PCR Whole-exome sequencing Whole-genome sequencing
Autosomal Dominant Polycystic Kidney Disease (ADPKD)	*PKD1, PKD2, HNF-1β, GANAB, DNAJB11*	Linkage analysis Direct mutation screening	Long-range PCR Whole-exome sequencing Whole-genome sequencing

Although linkage analysis helps identify markers that co-segregate with genes of interest, whilst also allowing for patient diagnosis confirmation in cases where mutation positions remain unknown^16^ and it can be effectively used for neonatal/prenatal testing and can identify the carrier status of at-risk females^17^, it also has limitations. Linkage analysis has got the risk of recombination, which could result in incorrect carrier status and the rise of *de novo* mutations/mosaicisms^16^ and it fails to provide details on the exact mutation type^17,18,19^. Another major difficulty associated with this technique is the requirement for multiple family members or generations for informative linkage analysis^16,19^, hence why direct mutation screening has become a more commonly practiced method of diagnosis.

Direct mutation screening is one of the most common and cost-effective methods used to diagnose Ciliopathies such as ADPKD and ARPKD, where the causative genes are known. The technique itself involves sequencing exonic regions of a particular gene, whilst flanking intronic regions, providing details about the mutation position and type^19^. Positively, samples for direct mutation screening are often easy to obtain with results being relatively definitive^16,19,20^. Furthermore, screening is now offered during neonatal and prenatal periods, making the technique more applicable to patients. In ARPKD, a definitive prenatal diagnosis can be carried out via direct mutation screening for disease-causing alleles after amniocentesis and chorionic villus sampling^21^. Unfortunately, this method of testing is only routinely offered to ‘at risk’ families, whilst the invasive nature of these methods also carries a risk of miscarriage at an approximate rate of 0.5–1%^21^.

It should be noted that direct mutation screening analysis can be useful in identifying isolated probands or in cases where *de novo* mutations arise but it can become quite restricting in cases where there are rarer mutations present or defects in other genes that are causative of the presenting phenotype^16^. For example, despite current knowledge regarding ADPKD, genes such as *HNF-1β*; *neutral α-glucosidase AB (GANAB)* and *DnaJ Heat Shock Protein Family (Hsp40) Member B11 (DNAJB11)* have recently been associated with presenting ADPKD-like phenotypes^22,23,24^. Similarly, mutations in *DZIP1L* have recently been associated with ARPKD with current efforts being made to identify other modifier genes in ARPKD^25^. Moreover, some phenotypes of cystic kidney disorders *e.g.,* ADPKD, ARPKD, NPHP and HNF1β-related disease may overlap (e.g. cystic expansion of the kidneys, kidney enlargement, liver fibrosis, *situs inversus*), making the identification of causative genes and mutations a complex and expensive process. Importantly, it can become complicated to sequence genes such as *PKD1* due to the presence of pseudogenes, which have a sequence homology of around 97.6–97.8%, making it challenging for the sensitivity of current diagnostic procedures to identify genuine *PKD1* regions^26^. In addition, in MKS, a highly heterogenous autosomal recessive ciliopathy that is lethal *in utero* or immediately after birth ^1,14,27^ and manifests a broad, multi-organ phenotype with considerable variations, key hallmarks of which include pulmonary hypoplasia and cystic kidney dysplasia ^1,4,5,14,27^, foetal sonographic abnormalities are detected in only ∼2.5% of pregnancies^28^, meaning that many cases will not be referred for direct mutation screening. Other techniques are also employed to detect known mutations, including Next Generation Sequencing (NGS) and quantitative PCR (qPCR) also known as, Real-time PCR (RT-PCR)^29^. When RT-PCR is selected as the molecular diagnostic technique in Preimplantation Genetic Diagnosis (PGD), TaqMan, which uses a labelled oligonucleotide (probe) with a fluorophore and a quencher probe at the 5′ and 3′ end respectively, is the most used assay^29,30^. The limited quantity of material available poses a great technical challenge in PGD, especially in cases of diagnosing heterogeneous disorders like MKS and in certain cases, fresh embryo transfer is necessary, typically 24 h after embryo biopsy to obtain a rapid genotype result^31^.

## “Evolved” PCR as a Method To Accurately Diagnose Ciliopathies

Advanced molecular diagnostic techniques in their entirety have evolved from the Sanger method, the “first-generation” DNA sequencing technique. Nowadays, in clinical practice, the Sanger method is used to validate NGS data, however, this additional level of assurance is not without fault, as it is costly, time-consuming, and not error-free^31^.

Multiplex qPCR expands on the advantages of RT-PCR, and it is a probe-based assay, where each probe is labelled with a unique fluorescent dye, allowing for simultaneous and rapid amplification of multiple genes in a single reaction^32^. Multiplex PCR is an ideal diagnostic technique for complicated heterogeneous Ciliopathies like ADPKD. ADPKD shows extensive allelic heterogeneity with six highly homologous sequences of *PKD1* exons 1–32, making molecular diagnosis complicated^26^. Multiplex qPCR was indispensable for detecting mutations in ADPKD patients that were overlooked by NGS^33^. In a study of 111 ADPKD patients, 86.6% of mutations were detected by NGS, however, one point mutation in exon 1 of *PKD1* and five deletions were overlooked by NGS and were only detected using multiplex qPCR^33^. For the six patients whose mutations went undetected, without multiplex qPCR, it is highly probable that they would have been provided a false negative result that could have led to delay in treatment^33^.

Another ciliopathy that exhibits similar molecular diagnostic complications to ADPKD is NPHP. NPHP is an autosomal recessive disease that leads to progressive renal failure and manifests as reduced kidney size, loss of corticomedullary differentiation and corticomedullary cysts, together with polyuria, polydipsia, anaemia, growth retardation and hypertension^1,14,34^. It relies on an initial clinical diagnosis via ultrasonography, followed by renal biopsy analysis, depending on the type of NPHP^34^. More than 25 genes have been associated with NPHP, with multiple NPHP genes being implicated with the onset of Joubert Syndrome and MKS^34^. In cases, where the most frequent mutation (*NPHP1*) is not identified as the pathogenic variant, Long-Read (LR) technologies that can detect repetitive regions of the genome *i.e.,* gene conversion events and are able to generate accurate assemblies^35^ are the preferred diagnostic method. Additionally, LR technologies can span both the low complexity and repetitive regions, meaning that they can detect paralogue regions of the genome, families of genes, and pseudogene homologs that are often overlooked by short read (SR) technologies^35^. It has been shown that LR PCR was successful in providing a more reliable ADPKD diagnostic result, when it targeted exons 1–32 of *PKD1*, generating amplicons from these regions and omitting pseudo-regions^26^.

The benefits of utilising LR technologies for not only diagnosing ciliopathies but other disorders outweigh the costs, which is often the main limitation associated with this technique. Nevertheless, over the past recent years, the cost has significantly declined, indicating that in the foreseeable future, more LR technologies will be utilised in clinical practice^35,36^.

## The Advances in Sequencing Technologies for Greater and More Accurate Data Capture in Ciliopathies

Targeted Exome Sequencing (TES), which concentrates on a specific panel of genes associated with disease pathogenesis and offers greater sequencing depth, reduced costs, and reduced data burden^37^, can comprehensively screen heterozygous carriers for a panel of known BBS genes, *i.e.,* 17 causative genes with a total of 242 coding fragments^38^ to identify pleiotropic disorders that have mutations in large genomic regions like in BBS.

Another option to advance current diagnostic practice for rare diseases is to implement Whole Exome Sequencing (WES). WES involves the sequencing of whole exomes in a genome and has improved sensitivity and efficiency, together with reduced costs^37^. 1–2% of DNA is comprised of exons, meaning that WES can analyse over 85% of all disease-causing mutations^39^. Implementing prenatal WES in suspected cases of MKS can help identify pathogenic variants^39^. In NPHP, a study has highlighted that using a combined approach of multiplex PCR and WES could help overcome the challenges of selecting the best candidate gene based on the phenotypic presentation of the disease^40^. Overall, WES could lead to an earlier and definitive diagnosis, aiding informed genetic counselling.

In some instances, Whole Genome Sequencing (WGS) which involves sequencing an individual’s entire genome, including coding and non-coding regions^41^ could be a more appropriate diagnostic method. For genetically heterogeneous disorders such as MKS, BBS, NPHP, the ability to concurrently sequence multiple disease-causing genes using WGS is crucial^41,42,43^. In ADPKD, WGS is capable of overcoming pseudogene homology complications, whilst in neonates with suspected ARPKD, STATseq, a method of WGS, can complete relevant data analysis within 26–50 h, allowing for rapid confirmation of disease^42^. Overall, the use of WGS dramatically eases pressure on clinicians and patients, whilst providing the potential for early diagnosis and aiding informative genetic counselling.

Using WGS is a straight-forward process that avoids the time-consuming procedure of targeted sequencing, allowing clinical professionals to identify all variant types in a single test^41,42^. This includes the detection of single-nucleotide variants, indels, large copy number variants as well as structural variants such as inversions and translocations^41^. In one ADPKD study, disease-causing variants were identified in 86% of patients using WGS, highlighting the effectiveness and informative potential of WGS as a diagnostic procedure^41,42^. Unfortunately, WGS has a relatively high cost, making it a more difficult diagnostic tool to access. However, recent advancements in the field, such as the use of Illumina HiSeq X sequencing system has reduced WGS costs^41^. In 2012, the 100,000 Genomes Project was launched in a bid to advance molecular diagnostics in the United Kingdom and enhance existing knowledge and research into cancer and rare disease^44^. The project is one of the first national health care systems that routinely uses WGS to sequence and identify any variations in around 85,000 National Health Service (NHS) patients, providing insight on disease aetiology, whilst advancing diagnostic and therapeutic prospects^44^.

## Conclusion

Attempting to identify the pathogenic variants within Ciliopathies, it is clear to see why advancements in molecular diagnostic techniques are undeniably needed. Current limitations lie with the time-consuming manner of diagnostic techniques due to the heterogeneity of Ciliopathies. The overlapping phenotypic presentations together with unclear molecular diagnostics in certain Ciliopathies pose limitations to techniques like direct mutation screening and linkage analysis. Although WES is more expensive than conventional Sanger sequencing, in the broader picture it is a more cost-effective tool. By employing WES as early as possible for diagnostic purposes, the financial burden on health services can be reduced, since multiple tests are not required to confirm the diagnosis, which can often also delay the start of treatment. Furthermore, disease management decisions can be based on a greater depth of information, increasing the reliability of the undertaken approach and informing accurate genetic counselling. Since WES has the capability of screening up to around 85% of a gene, it has a greater likelihood of identifying disease causing mutations than the currently employed techniques. Nevertheless, in diseases such as ADPKD, where pseudogene homology may pose complications in accurate genetic diagnosis, WGS would be the technique of choice. However, one of the major drawbacks of WGS is the cost and thus our suggestion is that WES became a commonly employed gold-standard technique in the field of biomedical science and diagnosis before further advancements to WGS.
